# Melanotic Pathology and Vertical Transmission of the Gut Commensal *Elizabethkingia* *meningoseptica* in the Major Malaria Vector *Anopheles gambiae*

**DOI:** 10.1371/journal.pone.0077619

**Published:** 2013-10-01

**Authors:** Idir G. Akhouayri, Tibebu Habtewold, Georges K. Christophides

**Affiliations:** Division of Cell and Molecular Biology, Department of Life Sciences, Imperial College London, London, United Kingdom; University of Crete, Greece

## Abstract

**Background:**

The resident gut flora is known to have significant impacts on the life history of the host organism. Endosymbiotic bacterial species in the *Anopheles* mosquito gut are potent modulators of sexual development of the malaria parasite, *Plasmodium*, and thus proposed as potential control agents of malaria transmission.

**Results:**

Here we report a melanotic pathology in the major African malaria vector *Anopheles gambiae*, caused by the dominant mosquito endosymbiont *Elizabethkingia* *meningoseptica* . Transfer of melanised tissues into the haemolymph of healthy adult mosquitoes or direct haemolymph inoculation with isolated *E* *. meningoseptica* bacteria were the only means for transmission and *de* *novo* formation of melanotic lesions, specifically in the fat body tissues of recipient individuals. We show that *E* *. meningoseptica* can be vertically transmitted from eggs to larvae and that *E* *. meningoseptica* -mono-associated mosquitoes display significant mortality, which is further enhanced upon *Plasmodium* infection, suggesting a synergistic impact of *E* *. meningoseptica* and *Plasmodium* on mosquito survival.

**Conclusion:**

The high pathogenicity and permanent association of *E* *. meningoseptica* with *An. Gambiae* through vertical transmission constitute attractive characteristics towards the potential design of novel mosquito/malaria biocontrol strategies.

## Introduction

The resident gut microbial flora is known to have significant impacts on the general physiology of the host organism that bears it [1,2]. Amongst metazoan, insect species contain a diversity of commensal microbial species interacting with the intestinal epithelium in distinct manners, from mutualism to opportunistic infections. Within the *Anopheles* -*Plasmodium* vector-parasite system, the involvement of the mosquito microbial flora in controlling malaria development was described decades ago, and current in-depth molecular mechanisms and identification of resident microbial species are gradually unveiled [3-12]. Malaria transmission relies on fertilisation and differentiation of *Plasmodium* within the mosquito gut, into motile zygotic stages i.e. ookinetes, which suffer major losses while dwelling for 18-24 hours in the gut lumen. Parasite losses are thought to be due to mosquito immune effectors [13-16], immune priming of the gut epithelium responding to the microbial flora [8,9] or direct anti-parasitic activities of resident species [11].

Culture-dependent and independent molecular analyses have led to the identification of the *Anopheles* microbial diversity from field-caught to insectary-derived mosquito colonies, and revealed the dominance in major malaria vectors of a few genera, including *Enterobacter* , *Pantoea* , *Pseudomonas* , *Serratia* , *Asaia* and *Elizabethkingia* [6,8,11,12,17,18]. The acquisition of most of these commensal bacteria by *Anopheles* mosquitoes remains unclear; however, *Asaia* *spp.* was shown to be vertically transmitted by egg-smearing in *An. gambiae* and *An. stephensi*, whereas *Elizabethkingia* *spp.* relies on horizontal and transstadial transmission [19-21].

In the present study, we investigated a chronic melanotic pathology in *An. gambiae* laboratory-reared mosquitoes and demonstrated that this is associated with the presence of the dominant gut commensal *Elizabethkingia* *meningoseptica* in the mosquito body cavity. We show that, in the absence of other gut flora, *E* *. meningoseptica* is highly virulent and synergizes with invading malaria parasite in rapidly killing the mosquito host. Finally, in addition to previous studies showing horizontal and transstadial transmission, we reveal that *E* *. meningoseptica* is enriched in mosquito ovarian tissues and can be transmitted to embryos. These features, i.e. pathogenicity and vertical transmission, could be utilised in the development of novel symbiont-based mosquito control strategies.

## Results

### Melanotic pathology throughout the mosquito development

**Figure 1:**
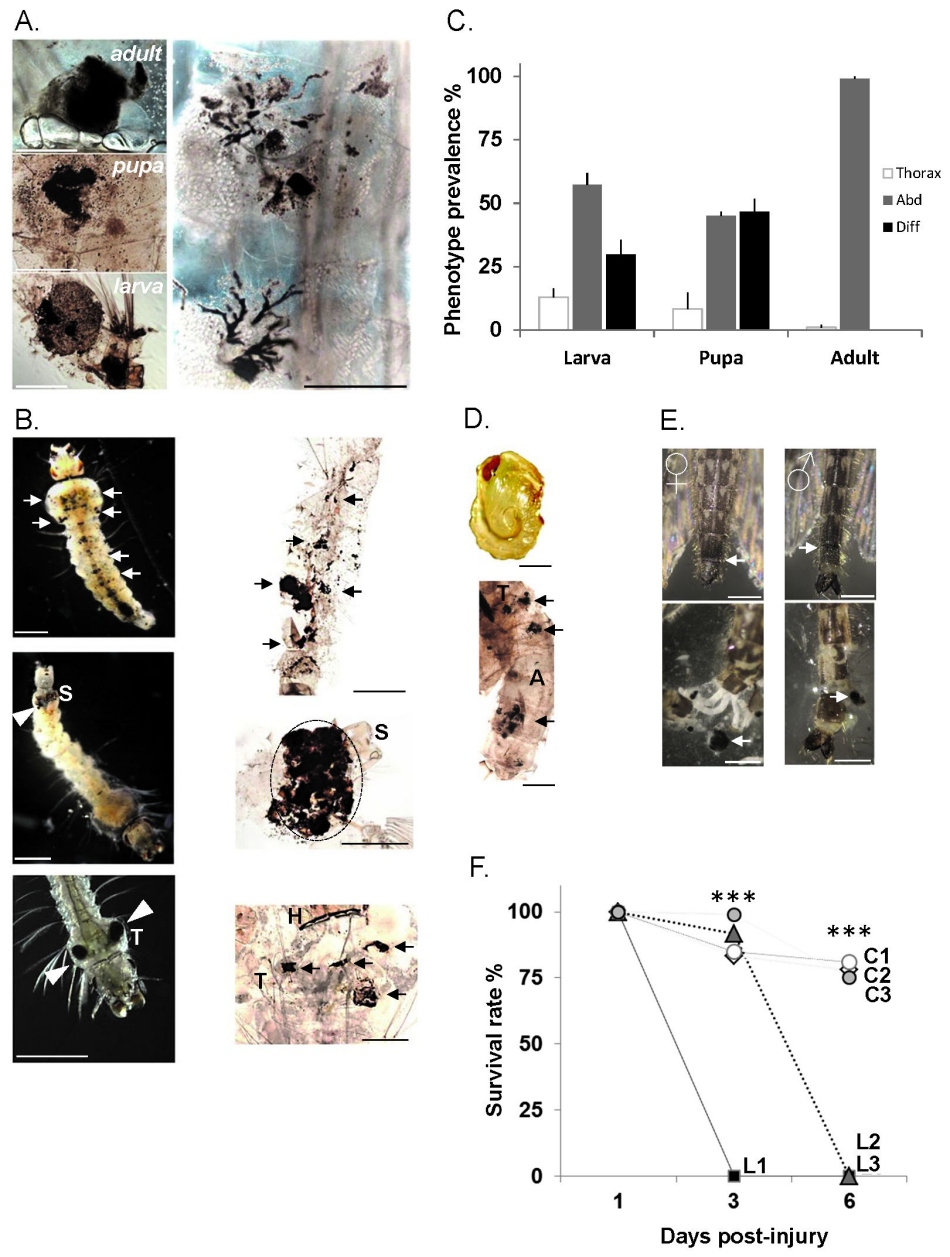
Melanotic phenotypes throughout *An* *gambiae* developmental stages. (**A**) Melanotic lesions in the mosquito fat body throughout the developmental stages (*left* *panels*; scale bar ~1mm). Variation in shape and size of lesions with clusters and multiple melanised cells in a trail-like progression (*right* *panel*; scale bar ~0.5mm) (**B**) Phenotypic larval lesions. Light micrographs (left panels) and fixed samples (right panels). Melanotic lesions diffused throughout the larval body (*upper* *panels*; arrows). Lesions specific to abdominal segments (*middle* *left* *panel*; white arrow head) visible through the cuticle below the siphon (S). Fixed abdominal segments showing complete melanisation of the 8^th^ segment (*middle* *left* *panel*; dotted area). Thoracic melanotic lesions (T) (*lower* *left* *panel*; white arrows), and a fixed larva with four lesions (lowest right panel) (black white arrows); H, head; T, thorax. Scale bar ~1mm. (**C**) Prevalence of melanotic phenotypes throughout the mosquito development is indicated as bars corresponding to the mean value +/- SD of 3 experiments. Abdominal, Abd; Diffused, Diff; Thoracic, Thorax. (**D**) Diffused phenotype in pupae. A light micrograph of a non-affected pupa (upper panel) and a fixed affected pupa with the diffused phenotype (lowest panel) showing melanotic lesions throughout the thorax (T) and abdomen (A) (black arrowheads). Scale bar ~1mm. (**E**) Lesions in adult mosquitoes. Melanotic abdominal lesions are observed at the junction between the 7^th^ and 8^th^ abdominal segments of female and male mosquitoes (*upper* *panels*; white arrows). Partially dissected abdominal segments from the same specimens (lowest panels) revealing melanotic aggregates (white arrows). Scale bar ~1mm. (**F**) Lesion-bearing mosquitoes survive poorly after thoracic injury. Groups of 40-60 female mosquitoes with lesions (L1, L2, L3) and corresponding controls without lesions (C1, C2, C3) were injured in the thorax below the base of the wing. Survival rate was monitored at 1, 3 and 6 days post-injury. Points indicated by *** are statistically very highly significant (P<0.05) between L and C.

A striking phenotype was observed in two independent laboratory colonies of the Afrotropical malaria mosquito *An. gambiae sensu stricto* (namely, the long-time colonised G3 strain and the more recently colonised *Ngousso* strain), whereby diseased individuals displayed melanotic aggregates underneath the cuticle at different stages of their lifecycle (Figure 1). Focusing on the G3 strain, this pathology was observed in over 10 generations at similar prevalence: 13.7% (+/- 6.7) in larvae, 12.4% (+/- 6.2) in pupae and 8.9% (+/-4.4) in adults, regardless of the mosquito density and rearing conditions (i.e. larval food, fructose solution for adults or rearing equipment handling). Mass counting was made out of 4 randomly chosen larval trays with over 2000 larvae per tray (2^nd^-4^th^ developmental instars), over 1000 pupae per tray and out of 3 cages with over 3000 adults per cage examined. Although being macroscopic observations, which do not exclude asymptomatic lesion carriers, these data suggested a stable association of mosquitoes and the putative lesion-inducing agent, which may have reach equilibrium between pathogen virulence and mosquito tolerance.

Dissection of affected individuals revealed that the lesions were specific to the fat body, regardless of life stage or phenotype distribution (Figure 1A, left panel). The severity of symptoms varied between individuals, but in most cases large melanotic aggregates with surrounding smaller foci were observed (Figure 1). In some instances, trails of melanotic cells could be noticed, inferring dissemination of the putative pathology inducing factor through fat body cells (Figure 1A, right panel).

Melanotic lesions could be classified into 3 distinct types according to their spatial distribution within the larval body cavity: diffused (Figure 1B, upper panels), abdominal (8th and/or 7th abdominal segments; Figure 1B, middle panels), and thoracic (Figure 1B, lower panels), the former two being the most prevalent types (Figure 1C). The pupal stage also showed distinct distribution of lesions with diffused and abdominal phenotypes predominating (Figure 1C, 1D). These two phenotypes did not appear to represent disease progression during development, but persisted without noticeable differences throughout the larval and pupal stages. The thoracic lesion phenotype was minor in larvae and pupae. In the adult mosquito, most if not all lesions were abdominal (Figure 1C, 1E), suggesting that throughout pupation the diffused phenotype was either removed or individuals with this phenotype died. Indeed, monitoring of diffused lesion-affected pupae revealed that they all died prior to adult emergence.

The insect fat body tissue is involved in critical metabolic processes, including resource storage, hormonal regulation and systemic immune responses. We therefore examined whether the melanotic pathology affects mosquito physiology. Although the life span of lesion-bearing mosquitoes was similar to healthy individuals (over 3 weeks in standard rearing conditions), thoracic injuries in these mosquitoes led to high mortality rates, as all mosquitoes died within 3 to 6 days post-traumatism, whereas healthy control mosquitoes fully recovered (Figure 1F). This suggests that the melanotic pathology reduces mosquito tolerance to injury.

### Induction of melanotic lesions and associated virulence

To gain a better understanding of the transmission mode of the melanotic pathology, we examined potential horizontal transmission by either feeding affected tissues to non-diseased individuals (i.e. larval water or fructose supplied to adults) or mixing lesion-bearing and lesion-free adults. None of the mosquito stages developed melanotic lesions suggesting that horizontal transmission is unlikely.

Since potential external sources did not induce lesion formation by ingestion or contact, we hypothesized that the lesions may develop by exposure of fat body tissues to the pathology factor within the mosquito body cavity. We therefore carried out transfer of melanotic fat body lysates via injections into the body cavity of non-affected adult mosquitoes.

**Figure 2:**
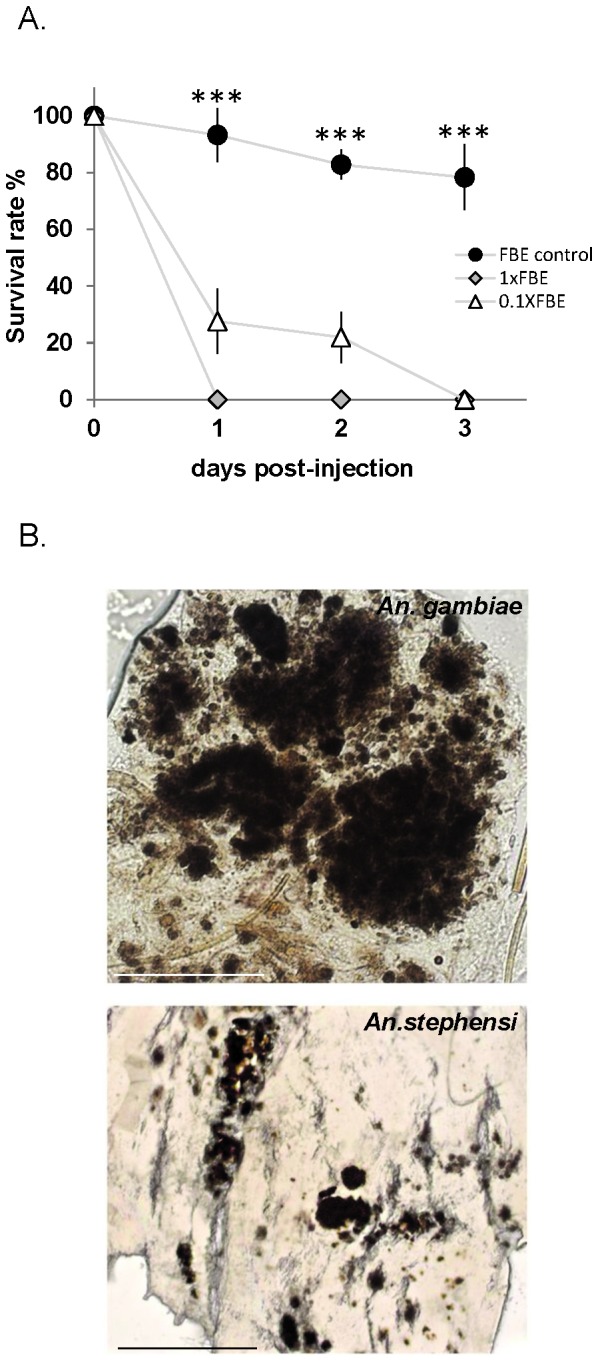
Induction of lesions by injection of melanotic tissues in *Anopheles* species. (**A**) Melanotic fat body tissues induce lethality in injected mosquitoes. Adult females were injected with abdomen-derived fat body tissues of melanotic lesion-bearing or of control adult females. Melanotic fat body extracts (FBE) were injected using two dilutions (1XFBE crude extract or 10-fold diluted extract 0.1XFBE). Survival rate was monitored daily, and represented as bars with standard error, as a percentage for each group. Three biologically independent injection assays were performed with 15-20 mosquitoes per condition. Points indicated by *** are very highly significantly different (P<0.05) between FBE control and 1XFBE or 0.1XFBE values.(**B**) Light micrographs of fat body tissues derived from FBE-injected female mosquitoes. Melanotic lesions in *An*. *gambiae* (*upper* *panel*; scale bar 0.05mm), and *An*. *stephensi* (*lower* *panel*; scale bar 0.5mm) mosquito species, resembling melanotic aggregates in the fat body of the affected ‘donor’ mosquito (see Figure 1).

Mosquito survival was strongly reduced upon transfer of affected tissues, while non-affected tissue injections did not have any impact on mosquito lifespan (Figure 2A). Importantly, mosquitoes injected with diseased tissues displayed *de novo* formation of melanotic lesions specifically in their fat bodies, suggesting direct transmission of potential pathogens (Figure 2B). The distribution of induced melanotic lesions was diffused throughout the mosquito body in more than 68% (+/- 5.13) of individuals, whereas 17% (+/- 1.28) showed a major aggregate at the point of injection and 20% (+/-1.5) died without apparent lesions, whereas injection of healthy fat body tissues did not have any impact on mosquito life span, even at the highest concentration (Figure 2A). A similar induction of lesions was observed in the Asian mosquito vector *An. stephensi* suggesting broad specificity of the putative pathology factor to mosquitoes (Figure 2B).

We examined whether antibiotic treatment applied to the aquatic larval environment affects the prevalence of the pathology phenotype. The use of a cocktail of penicillin and streptomycin did not affect the overall phenotype prevalence in larvae (Figure S1). A noticeable antibiotic effect was a developmental delay indicating a tight relationship between the mosquito development and the bacterial flora present in the breeding environment (Figure S2). However, treatment of mosquito embryos with an antiseptic solution (0.3% Virkon® v/v) with large antimicrobial spectrum against fungi and bacteria dramatically reduced the phenotype prevalence in larvae from approximately 20% to a near complete absence; one affected individual was observed in over 3000 larvae, suggesting vertical transmission from adult female mosquitoes to embryos. Resulting adults from treated eggs did not show any lesions.

Taken together, these results led us to hypothesize the involvement of causative microorganisms in the observed fat body pathology through embryo contamination.

### Characterization of *Elizabethkingia* *meningoseptica* as the causative agent

Plating of melanotic fat body tissue homogenates in rich-medium agar plates revealed the presence of white/yellow bacterial colonies that were absent from control tissues within the limits of our extraction and plating procedures.

Gram and Giemsa staining revealed that all colonies were gram-negative rod-shaped cells (Figure S3) that were highly resistant to ampicillin and less albeit also resistant to penicillin/streptomycin treatments.

We carried out sequencing of 16s ribosomal DNA from several of these isolates using degenerate primers and in all cases identified *Elizabethkingia* *meningoseptica* as the only bacterial species present, with sequence identity up to 99% with *E* *. meningoseptica* strains and 98% with the previously isolated *An. gambiae* midgut-derived strain *E* *. anophelis* [22,23]. *E* *. meningoseptica* is a known multi-antibiotic resistant species, first isolated from infants suffering from meningitis, and causing nosocomial infections in neonates and immune-compromised patients [24,25]. Interestingly, this bacterium was also frequently described as a dominant gut endosymbiont of both field-caught and laboratory-reared anophelines [8,12,17,20,26,27].

**Figure 3:**
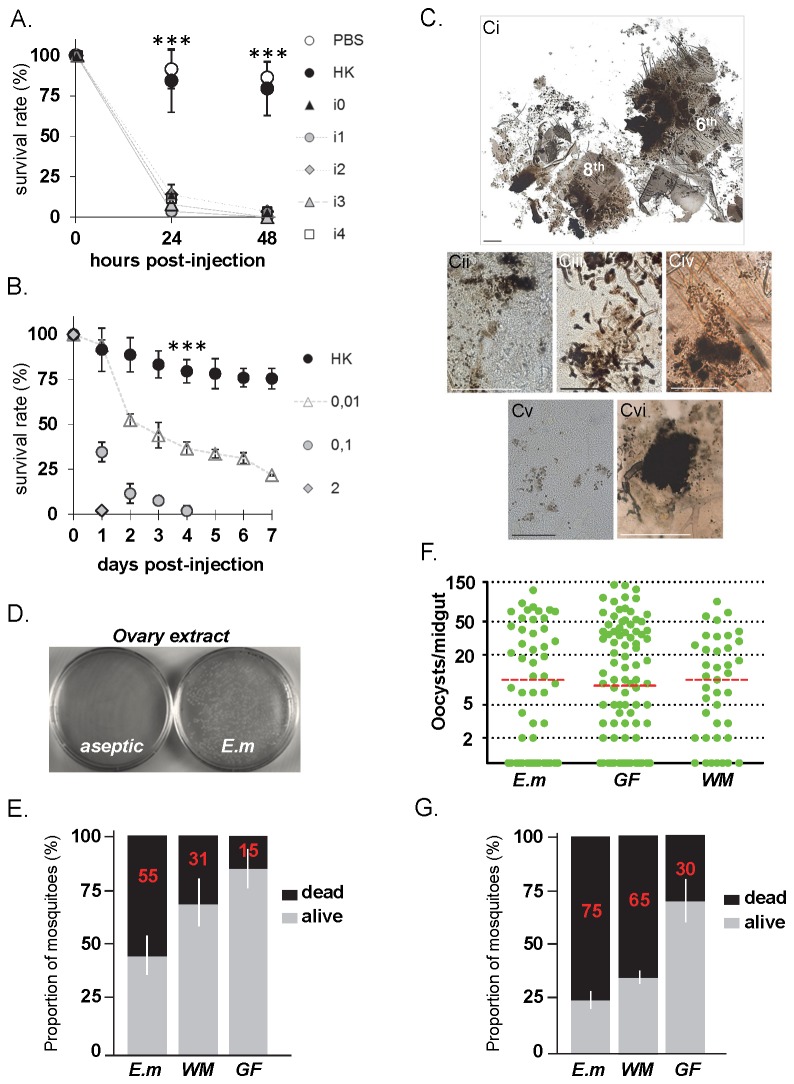
Pathogenicity, lesion induction and vertical transmission of *E* *. meningoseptica* *.* (**A**) Virulence of *E* *. meningoseptica* toward mosquitoes. Independent isolates of *E* *meningoseptica* (isolates 0 to 4) were injected in adult females. Survival rate is shown as percentage with standard error (n=120 per isolate) from 3 independent assays. Sterile PBS and heat-killed (HK) bacterial cultures were injected as controls. Points indicated by *** are statistically very highly significant (P<0.05) between HK and isolate injected samples. (**B**) Mosquito survival rate after injection of serial dilutions of *E* *. meningoseptica* (isolate 0) as indicated; n=180 from 3 independent assays. Values of HK and live bacterial samples were very highly significantly different (P<0.05). (**C**) Induced melanotic lesions in the fat body by *E* *. meningoseptica* ; (Ci) Partially dissected 6-8^th^ abdominal segments after bacterial injection showing massive melanisation of fat body tissues; (Cii) Dissemination of melanotic spots throughout the abdominal fat body; (Ciii) Trail-like melanisation; (Civ) An aggregate with disseminated spots; (Cv) Melanised individual fat body cells (Cvi). A major melanotic thoracic aggregate. Scale bar 0.5mm (Ci, Ciii-Cvi), 0.2mm (Cii). (**D**) Enrichment of *E* *. meningoseptica* in *An*. *gambiae* ovaries. *E* *. meningoseptica* fed to germ-free females shows colonization of ovaries (E.m), whereas aseptic adults did not show any colony. (**E**) Mosquito lethality upon bacterial recolonisation. *E* *. meningoseptica* (E.m) or whole microbiota (WM) suspensions were fed to germ-free mosquitoes (GF) and survival was assessed 24h later. Non-supplemented GF mosquitoes were used as control. Bars show distribution as percentage (mean value in red with standard error) of dead (black area) and alive (grey area) mosquitoes (n=80) from 2 independent experiments. (**F**) *Plasmodium berghei* development is not affected by bacterial reconstitution. E.m, WM and GF mosquitoes were infected with malaria 24h post-reconstitution, and oocyst load monitored 7 days post-infection, represented as oocyst number per midgut (green dot) with mean values as red dotted bar. Mosquitoes were blood-fed on the same infective host (n=120) from 2 independent experiments. No statistical difference was found in between all oocyst load mean values. (**G**) Synergistic effect on mosquito survival upon bacterial reconstitution and *Plasmodium* infection. Mosquito survival of malaria infected-Em, -WM and -GF was assessed 24h post-infection.

We assessed the association of the isolated *E* *. meningoseptica* bacteria with the observed melanotic pathology by direct injection of bacterial suspensions into the body cavity of adult female mosquitoes. Mosquito survival was severely affected following injection with live *E* *. meningoseptica* , while injection with heat-inactivated bacterial suspension had limited impact on the mosquito lifespan (Figure 3A, 3B). Dissection of live bacteria-injected mosquitoes revealed the presence of numerous melanotic aggregates as well as single melanised cells throughout their body cavity (Figure 3C), whereas control mosquitoes (injected with heat-inactivated bacteria suspension or PBS) did not present any melanotic lesions even after death.

These data suggested that the melanisation pathology observed in bacteria-injected mosquitoes is a direct consequence of *E* *. meningoseptica* infections. The impact of *E* *. meningoseptica* infection on mosquito survival rates and melanotic lesion development in fat body tissues was reproduced with all available adult- and larval-derived isolates fully substantiating our findings and conclusions.

### Vertical transmission of *E* *. meningoseptica*

The chronic melanotic pathology associated with *E* *. meningoseptica* in the *An. gambiae* mosquito colony prompted us to investigate a potential vertical transmission from females to offspring.

We generated *E* *. meningoseptica* -mono-associated adult females from the affected G3 colony by treating mosquitoes with an antibiotic cocktail of penicillin, kanamycin, streptomycin and ciprofloxacin administered with the fructose solution, mosquitoes being germ-free at this stage by checking microbial depletion of their gut extracts plated on nutrient media (not shown). We then reconstituted their gut flora with *E* *. meningoseptica* or whole microbiota. We first confirmed efficient invasion and establishment of *E* *. meningoseptica* in the mosquito gut by aseptic dissection of guts from *E* *. meningoseptica* -mono-associated adult females and plating on nutrient media (Figure S4). *E* *. meningoseptica* -mono-associated females were then blood-fed and their ovaries were dissected. Strikingly, a high enrichment of *E* *. meningoseptica* was observed in mosquito ovaries (with more than 4.10^4^ colony forming units per pair of ovaries) (Figure 3D). F1 Larvae derived from the *E* *. meningoseptica* -mono-associated females also showed high abundance of the bacterium (Figure S4). Microbial analysis on conventionally-reared larvae raised in the very same aquatic environment as *E* *. meningoseptica* -mono-associated-F1 larvae, showed high bacterial diversity with some instances of *E* *. meningoseptica* not being detected (Figure S5). Moreover, *E* *. meningoseptica* -reconstituted F1 larvae solely harbour *E* *. meningoseptica* , whereas they were exposed to the same water-borne microbial diversity (Figure S5).

Taken together, these results indicate, on the one hand, a poor microbial acquisition from the rearing water, and on the other hand, an efficient vertical transmission of *E* *. meningoseptica* by ovarian infection and subsequent transmission to embryos and larvae, which may explain the persistence of the melanotic pathology associated with *E* *. meningoseptica* .

### Negative synergistic impact of *E* *. meningoseptica* and *Plasmodium berghei* on mosquito survival

Next, we investigated the impact of *E* *. meningoseptica* on adult mosquito survival using three groups of adult female mosquitoes: germ-free mosquitoes (GF), antibiotic-treated mosquitoes of which the gut flora was reconstituted (WM) with facultative or obligate aerobic bacteria prepared from gut extracts of 3-day old females, and *E* *. meningoseptica* -mono-associated mosquitoes (*E.m*). *E. m* mosquitoes showed a high mortality rate of 55% within 24h post-reconstitution, whereas WM mosquitoes had a 31% mortality rate (Figure 3E). The mortality on GF mosquitoes was 15%. These data suggest that virulence of *E* *. meningoseptica* upon feeding is higher in the absence of other gut commensals. To assess the impact of *E* *. meningoseptica* on *Plasmodium* development, we carried out infections of *E. m*-, WM- and GF-mosquitoes with the rodent malaria parasite *Plasmodium berghei*. The intensity of infection was similar between all mosquito groups, suggesting that *E* *. meningoseptica* does not have a significant impact on *Plasmodium* development in the mosquito gut (Figure 3F). However, bacterial reconstitution led to high mortality in WM-reconstituted mosquitoes, which was further enhanced in infected-*E*.*m* mosquitoes (Figure 3G). These data may indicate a synergistic negative impact of this dominant gut bacterium, as well as whole microbiota and *Plasmodium* on mosquito survival, which is in line with previous studies showing that co-infections with *Plasmodium* and bacteria result in increased mosquito mortality [8,9].

## Discussion

Here, we report that *E* *. meningoseptica* , a major *Anopheles* gut endosymbiont, is highly virulent to adult *An. gambiae* mosquitoes and is associated with chronic melanotic lesions of fat body tissues. We show that transfer of *E* *. meningoseptica* or melanotic tissues in lesion-free mosquitoes trigger *de novo* formation of lesions specifically in the fat body tissue, fulfilling Koch’s postulate of reproducibility of disease in otherwise non-affected individuals.

Moreover, this is the first report on induction of tissue melanisation by a bacterial infection in mosquitoes.

Melanisation represents one of the major immune defence responses unique to arthropods, leading to the production of melanin and its deposition on the surface of invading microorganisms, as well as being involved in wound healing, egg hardening and cuticle pigmentation [28,29]. It can be a cellular–mediated process induced by immune cells in response to injury or to the presence of a parasitic agent, or humoral responses through pathogen recognition and activation of the prophenoloxidase pathway [30-33]. Whether the melanotic pathology is due to a mosquito melanisation response against this virulent bacterium or the bacterium inducing tumour formation and melanisation specifically in fat body cells remains to be investigated.

It has been previously shown that non-gut resident gram negative *Escherichia coli* or gram positive *Staphylococcus aureus* bacteria injected into the mosquito body cavity are melanised [34,35]; however, this reaction does not spread to host tissues. These, in conjunction with the finding that live *E* *. meningoseptica* are present in melanotic lesions and can induce tissue melanisation upon transplantation, support the latter hypothesis.

Melanotic lesions, intuitively referred to as melanotic tumours or metastases, have been previously reported in insects [36]. Development of human malignant melanoma and formation of “tumor” like or melanotic aggregates in insects showed striking similarities in term of biochemical steps involving enzymatic reactions leading to melanin production [37]. In the fruitfly *Drosophila melanogaster*, mutants generated by random mutagenesis or through targeted gene disruption display melanotic tumours and transplantation experiments triggered lesions in recipient tumour-free individuals [38-41]. A wide range of melanisation phenotypes was observed in several insect species where major insect immune pathways were over-activated [42,43].

Compiled microbiome descriptions in *Anopheles* mosquitoes clearly reveal the strong symbiotic nature of *E* *. meningoseptica* , which has been isolated from several independent sources including semi-field *An. gambiae* females in Kenya, field sampled mosquitoes in Cameroon [12,44] and laboratory colonies where it is the predominant species [8,12,20]. Moreover, although rearrangement of the microbial communities occurs after mosquito bloodfeeding, *E* *. meningoseptica* remains a dominant resident bacterium in both blood-fed and non-blood-fed females [26,27]. Given the dominance of only a few genera in the mosquito microbiome, it has been suggested that a core gut microbiota may be associated with *Anopheles* mosquitoes [12], to which *E* *. meningoseptica* may belong.

Impact of commensal species within the microbiome community on insect species is relatively unknown. A few studies give details on specific species regarding their impact on host life traits. Isolation of bacterial symbionts from field-collected *Anopheles* *arabiensis* has identified a gut resident *Enterobacter* with anti-plasmodial activities both *in vitro* and *in vivo* due to production of reactive oxygen species [11]. Studies on germ-free *versus* conventionally-reared insects have shed light into numerous physiological interactions between the host and commensal bacteria, especially host development, immune tolerance and protection from pathogenic infections [45-49]. Notably, dysregulation of the gut homeostasis in *D. melanogaster* by altering bacterial communities has revealed a cryptic resident bacterium, *Gluconobacter* *spp.* (G707), which in the absence of antagonist bacteria over-proliferates triggering gut cell apoptosis and eventually host death [46]. Fly lethality was prevented by supplying beneficial commensals counteracting G707 virulence. Similarly, we have shown that, by supplying *E* *. meningoseptica* to bacteria-free mosquitoes, these had compromised survival compared to whole microbiota-reconstituted individuals, suggesting that bacterial community shift toward *E* *. meningoseptica* proliferation in the mosquito gut exacerbates *Elizabethkingia* virulence.

It is noteworthy to mention that, one the one hand, *E* *. meningoseptica* is widespread in mosquitoes, and on the other hand, is able to cause human diseases [8,12,25,27]. This suggests that this bacterial species is able to inhabit diverse niches and is clearly well-adapted to mosquito gut environments.

Historically, vector control remains the most cost-effective and health-efficient measure against disease transmission [50,51]. Besides current and efficient insecticide-based control programs [52-54], alternative approaches such as vector biocontrol have been encouraged to anticipate and mitigate the emergence of insecticide resistance [55,56]. In addition, insecticide susceptibility and susceptibility to microbial agents have been frequently shown to negatively correlate, suggesting that biocontrol, although not yet assessed at a large scale, could become part of malaria integrated management control programs [57,58].

Microbial symbiont-based strategies are a novel approach toward vector control [44,59-65], and can be divided into two main categories: vector population suppression exploiting symbiont virulence and paratransgenesis, whereby symbionts are genetically modified to express molecules that render the host refractory to pathogen transmission [11,17,44]. In view of malaria transmission control using mosquito commensals, such microorganisms should display either of two broad characteristics, respectively: a degree of virulence towards the mosquito host that is either inherent or genetically engineered and/or permanent association with the targeted vector population, ideally through vertical transmission or through prompt acquisition from the environment [6,60,65]. To date, three bacterial genera, *Asaia*, *Panteoa* and *Wolbachia* , fulfil these critical parameters. Indeed, proof-of-principle paratransgenesis approaches have been validated in *An. gambiae* and *An. stephensi* using the two vertically-transmitted symbionts, *Asaia* *spp.* and *Pantoea* *agglomerans* [19,44]. *Wolbachia* stable association with *Anopheles* *stephensi* was recently established [66]. Our discoveries about vertical transmission, stable host association and virulence in mono-association with mosquitoes make *E* *. meningoseptica* an attractive candidate symbiont to be added to this list. Modulating the bacterial community in the *Anopheles* gut in order to favour pathogenic species, such as *E* *. meningoseptica* may lead to high mortality in the mosquito host. The high prevalence of *E* *meningoseptica* in several disease vector mosquitoes is an additional advantage in view of developing control approaches that require constant contact and high abundance of the symbiont in the target species.

## Materials and Methods

### Ethics statement

This study was carried out in strict accordance with the United Kingdom Animals (Scientific Procedures) Act 1986. The protocols for maintenance of mosquitoes by blood feeding and for infection of mosquitoes with *Plasmodium berghei* by blood feeding on parasite-infected mice were approved and carried out under the UK Home Office License PLL70/7185 awarded in 2010. The procedures are of mild to moderate severity and the numbers of animals used are minimized by incorporation of the most economical protocols. Opportunities for reduction, refinement and replacement of animal experiments are constantly monitored and new protocols are implemented following approval by the Imperial College Ethical Review Committee.

### Mosquitoes

Mosquito colonies of *An. gambiae sensu stricto* strains G3 colonised for decades (http://www.mr4.org/) and *Ngousso* recently colonised, and *An. stephensi* Liston were reared at a temperature of 27-28°C and 70-75% relative humidity with a 12-hour light/dark cycle. Larvae were reared in plastic trays filled with 0.1% salt water (w/v) and fed daily on ground cat food. Pupae were collected and pooled from approximately 10-15 larval trays. Emerging adults were fed *ad libitum* on a 10% fructose solution in sterile water (w/v).

### Phenotypic characterisation

Screening of lesion-bearing individuals was carried out visually in larval trays for larvae (estimated n=2000 per tray, with 4 rearing trays examined) and pupae (n=1000 per tray, with 4 rearing trays examined), and under light microscopy for adults after slight immobilisation on ice (n=3000 per cage, with 3 cages examined). In some instances, lesion-bearing individuals were washed in sterile PBS and fixed in 4% paraformaldehyde for 30 min. Once washed in PBS, samples were mounted in 50% glycerol (v/v water) on glass slides and visualized under light microscopy.

### Transmission assays in embryos and larval stages

In an attempt to trigger the melanotic phenotype in non-diseased individuals, melanotic fat body tissues were dissected from 10 *An. gambiae* adult females harbouring the abdominal lesion phenotype, and supplied to embryos and larval stages of an independent *An. gambiae* colony that did not present lesion-bearing individuals throughout the mosquito development. Fat body tissues were homogenised in a minute amount of PBS using a plastic pestle to obtain the fat body extract (FBE).

#### Embryos

Gravid females (n=2-3) were forced to lay their eggs (n>30) onto FBE resuspended in 100 µL 0.1% salt water in 1.5 mL microfuge tubes for 30 min. Larvae were observed on the day of hatching or the day after under light microscopy. The remaining larvae were transferred to a 90 mm-diameter petri dish, and allowed to develop further into pupae, which were observed under light microscopy for presence of lesions.

#### Larvae

II^nd^ and III^rd^ instar larvae (n=50-60) were placed in a droplet of FBE resuspended in 1 mL 0.1% salt water in a 90 mm-diameter petri dish, and observed daily with appropriate supply of water and food, until pupation. The pupae were then visualized.

### Transmission assay of melanotic lesions in the adult mosquito stage

All infection and injection assays were performed with newly emerged or 1 day old *An. gambiae s.s.* G3 females with melanotic lesions and non-diseased females from an independent G3 colony without any lesions.

#### Thoracic Injury

Adult females either harbouring the melanotic abdominal phenotype or non-affected (collected from the non-diseased colony) were pricked below the base of the wing, with a microcapillary glass needle. Groups of 40-60 females in triplicates were used, and survival rate post-injury was monitored.

#### Adult feeding

A 1 mL FBE solution (as described above) was mixed 1:1 with 10% fructose solution (w/v), and provided to non-affected adult females on a cotton pad. Adults were allowed to feed for 4-5 days and were partially dissected to expose and observe fat body tissues.

#### Injection of fat body extracts

Lesion-free individuals were injected with ~37 nL of FBE resuspended in 10 µL sterile PBS, in the thorax using microcapillary glass needles and a nano-injector (Nanoject II, Drummond Sci.). Different FBE concentrations were injected into 2 different groups of mosquitoes: 1X FBE.µL^-1^ for Group 1 (n=45 over 3 independent assays); 0.1X FBE.µL^-1^ for Group 2 (n=60 over 3 independent assays). Control specimens (n=45) were injected with 0.1X FBE, with FBE produced from abdomens of non-affected females. *An. stephensi* mosquitoes from a distinct insectarium facility were also injected with 0.1X FBE. Mosquitoes were immobilised with a constant CO_2_ flux during the procedure, and were immediately returned to the insectarium after injection. Injected mosquitoes generally recovered within 20 min, and remained under insectarium conditions until collection for phenotypic analysis. Survival rate post-injection was monitored regularly and dead individuals were promptly dissected to verify the presence of lesions. Dissected mosquitoes were then classified into three categories according to the distribution of melanotic lesions observed: diffused throughout the mosquito fat body, aggregated in the thoracic area, or no apparent lesions.

### Antimicrobial treatment of mosquito stages

#### Larvae

Antibiotic treatment of *An. gambiae* larval stages was carried out using a solution of penicillin-streptomycin (5000 units-5000 µg.mL ^-1^, Gibco) as follows: I^st^ to III^rd^ larval instars collected from rearing pans containing melanotic lesion-bearing individuals, were submerged in two antibiotic dilutions (25 mL in 1 L of 0.1% salted water [Full antibiotic condition]; 5 mL in 1 L [Partial antibiotic condition]). The daily collection of lesion-bearing larvae, as well as the counting of moulting larval instars, pupae and emerging adults was conducted.

#### Embryos

Embryos originating from the lesion-bearing colony were soaked in 0.3% Virkon® in salt water (w/v), a potent antimicrobial agent for 3 minutes. Treated embryos were rinsed several times in water, and then maintained under standard conditions (as stated above), and the visual observation of any lesion-bearing individuals was carried out daily throughout the mosquito development.

### Bacterial isolation and infection assays

#### Microbial isolation

To isolate endogenous mosquito microorganisms from the melanotic lesions, two independent pools of 10 *An. gambiae* adult females harbouring the abdominal phenotype were briefly immersed in absolute ethanol and sterile PBS, and their melanotic fat body tissues were dissected as described above, in sterile conditions. Two additional independent extractions using 4^th^ instar larvae displaying the diffused melanotic lesions, were also processed; larvae were washed in sterile PBS, briefly immersed in absolute ethanol and washed in PBS once more. Slight opening of abdominal segments without disruption of the gut was carried out on a droplet of sterile PBS. Collection of fat body tissues was performed by careful pipetting near the wound, and homogenisation of tissues in 100µL sterile PBS.

The melanotic tissues were plated onto LB agar for standard bacterial growth, and malt-agar supplemented with peptone. Non-melanotic fat body tissues from adult females and larvae were used as controls, and treated as above. Plates were incubated at room temperature until the appearance of colonies. Yellowish-white colonies specific to melanotic tissues were further used in transmission assays.

Five randomly selected lesion-specific individual colonies (1 derived from adult, and 4 derived from larvae affected with the diffused phenotype) were inoculated in LB broth and grown overnight at 37°C on a shaker. Aliquots of bacterial cultures pre-treated for 2 h with ampicillin (10 mg/mL) or penicillin/streptomycin (5000 units-5000 µg.mL ^-1^) were plated onto LB agar plates, and incubated overnight at 37°C to test for antibiotic susceptibility.

Gram staining was carried out. Briefly, bacterial aliquots were smeared and allowed to dry. Samples were successively covered with crystal violet for 1 min, washed with water, immersed in iodine solution for 1 min, washed with water, and decolorized with acetone-alcohol solution. Samples were then covered with safranine for 1 min and washed with water before microscopic observation. All solutions were purchased from Sigma-Aldrich.

Giemsa staining for morphological observations was carried out by brief methanol fixation of a droplet of bacterial culture on glass slide, and incubated for 5 min in Giemsa solution, prior to wash in water.

#### Bacterial DNA extraction and species identification

For genomic DNA extraction, yellowish-white individual colonies randomly selected were inoculated in LB liquid for 4h at 37°C. Cells were pelleted and washed once with sterile PBS. Genomic DNA extraction was carried out using the Wizard® Genomic DNA Purification Kit (Promega), according to manufacturer’s instructions. Purified genomic DNA was resuspended in 50µL sterile water prior to PCR amplification.

16S rRNA gene specific primers, 1492r and 27f as described in [67] were used to amplify partial sequences (>750bp) of 16S ribosomal gene from bacterial isolates.

PCR products were gel-extracted using a Qiaquick Gel extraction kit (Qiagen) according to manufacturer’s instructions. Sequencing of the purified PCR products was conducted at Natural History Museum, London using the PCR primers. ClustalW and NCBI blast softwares were used to recover 16S rRNA sequences from the sequenced isolates, resulting in the identification of *Elizabethkingia* *meningoseptica* . Additionally, conventionally-reared larvae raised in the same water as individuals derived from *Elizabethkingia* -reconstituted females, were processed as described above, and 16S PCR, sequencing and Blast alignments identified *Asaia* *sp.* *,* *Cedecea* *sp.* *,* *Elizabethkingia* *sp.* *,* *Gibbsiella* *sp.* *,* *Klebsiella* *sp.* *,* *Plesiomonas* *sp.* *,* *Rahnella* *sp.* and *Serratia* *sp* (Figure S3).

#### Bacterial injection in adult mosquitoes

Using five randomly isolated *E* *. meningoseptica* colonies, overnight bacterial cultures [LB supplemented with ampicillin (10 µg.mL ^-1^); 37°C] were twice pelleted at 5,000*g* for 15 min, and resuspended in sterile PBS to appropriate concentrations i.e. optical density at 600nm (OD_600nm_) described on figures. Groups of 40-60 adult females per bacterial concentrations were injected, as described above for FBE injections. Sterile PBS and heat-inactivated (10 min incubation at 95°C) bacterial cultures were used as controls. The heat-inactivated solution contains equal volumes of each isolate at OD 0.1. Survival rate post-injection was monitored daily. Both dead and living mosquitoes were processed for tissue dissection and microscopy.

#### Bacterial transmission assays

For oral infections, a culture grown overnight at 37°C was pelleted and adjusted to O.D._600nm_ 2 in sterile PBS. Embryos and larvae were submerged in the bacterial culture, and adults were fed the culture with 10% fructose on a cotton pad supplied twice daily with bacterial culture to ensure constant exposure and feeding. All stages were monitored for 2 weeks prior to the dissection and microscopic examination of fat body tissues.

### Bacterial reconstitution and malaria infection

#### Whole microbiota and *E* *. meningoseptica* reconstitution

Newly emerged *An. gambiae* females were supplied with an antibiotic cocktail of penicillin (10 units/mL), kanamycin (25 mg/mL), streptomycin (25 mg/mL) and ciprofloxacin (25 mg/mL), mixed in sterile 10% fructose solution. Antibiotics were provided for 2 days and added twice daily on a cotton pad for constant exposure to mosquitoes. Once antibiotic removed, mosquitoes were supplied for 24h with sterile fructose solution to wash out antibiotic remnants. A culture of *E* *. meningoseptica* or total cultivable midgut-derived bacteria (O.D_600nm_=15) isolated from 3-days old females were supplied to antibiotic-treated mosquitoes (n=40 per experiment from 2 biologically independent assays) in sterile fructose solution for 24h. Surviving/dead mosquitoes were counted 24h post- reconstitution to assess impact of gut recolonisation with bacteria.

#### *Plasmodium* infection and mosquito survival post-infection

Antibiotic-treated mosquitoes reconstituted with *E* *. meningoseptica* or whole microbiota (n=60 surviving mosquitoes per experiment from 2 biologically independent assays), were either fed with *Plasmodium berghei*-infected blood meal 24h after reconstitution. The mosquito survival post-malaria infection was monitored 24h post-blood meal, when malaria ookinetes develop and breach through the mosquito gut epithelium For *Plasmodium* -fed mosquitoes, oocyst load in the mosquito midgut was assessed 7 days post-infection in antibiotic-treated non-reconstituted (i.e. germ-free), whole-microbiota- and *E* *. meningoseptica* -reconstituted mosquitoes in two biologically independent infection experiments.

### *E* *. meningoseptica* colonisation of mosquito tissues and vertical transmission

Germ-free mosquitoes were produced as described above. In order to determine the efficacy of reconstitution and the transmission mode of *Elizabethkingia* in the mosquito, 10 individual *E* *. meningoseptica* -reconstituted females were surface-sterilised by successive washes in 75% ethanol and sterile PBS, and their midguts were dissected in aseptic conditions. Pooled gut extracts were homogenised with a sterile pestle in liquid LB medium, and spread on ampicillin-containing LB agar. Plates were incubated at 37°C, and colony forming units were counted 24h after extract plating. 10 additional females were allowed to feed on blood and kept for 3 days on sterile 10% fructose solution until aseptic dissection, serial dilution and plating of ovaries, as described for midgut processing.

Moreover, a set of blood-fed females (n=5 from 2 biologically independent assays) was allowed to egg-lay in sterile petri dishes lined with filter paper impregnated with water supplied with an antibiotic cocktail (as described above). Eggs were left to hatch in the petri dish and, surface-sterilised one day-old larvae were homogenised in liquid LB. Extracts were plated on solid LB agar containing ampicillin and maintained at 37°C. Colony forming units were quantified 24h after plating. In all plating, colonies were processed for 16S PCR identification, which, in all cases (midgut/ovary/F1 larvae) identified *E* *. meningoseptica* .

## Supporting Information

Figure S1 **Antibiotic treatment does not affect recovery of lesion-bearing larvae.** Antibiotic solutions were applied to larval stages 7-8 days post-egg laying (n=300), from 3 biological replicates. Larval instars harbouring melanotic lesions were counted daily until pupation (None). Normal diet without antibiotics; (Partial) Intermediate antibiotic concentration [5mL streptomycin/Penicillin (200X dilution in 1L rearing water)]; (Full) Concentrated antibiotic treatment [25mL Pen/Strep (40X dilution in 1L rearing water)]. Mean value of relative numbers of larvae showing lesions is represented as bar with standard for each condition.
(TIFF)

Figure S2 **Mosquito developmental delay after antibiotic treatment.** Antibiotic treatment was applied to larval stages as described in Figure S1 (*black line*). Control normal diet without antibiotics; (*dark grey*) Partial treatment; (*light grey*) Full treatment. (**A**) Adult emergence; Note that most adults appeared at day 9 in control, whereas antibiotic-treated groups showed 65-70% emergence on the same day. (**B**) Pupation peaks at day 4 in normal diet, whereas a 5 days-delay occurred in treated groups for pupation to proceed. (**C**) Larval developmental rate; a severe developmental slow-down appeared in treated larvae, since 50% of all moulting (from I^st^ to IV^th^ instars) occurred 4-5 days later compared to controls, showing the strong impact of antibiotics at the larval feeding stages.
(TIFF)

Figure S3 **Identification of *E* *. meningoseptica* .** Micrographs of ampicillin-LB plates with bacterial colonies derived from control (*Healthy*; top left panel) and lesion-affected (*Lesion-derived*; *top* *right* *panel*) fat body tissues from larvae. Note major bacterial colonies (white arrows) specifically recovered from lesion-affected tissues. These colonies were Gram-stained-negative rod-shaped cells (*Gram*; *lower* *left* *panel*) with individual (black arrows) and dividing cells (white arrowheads) visible on Gram- and Giemsa-stained bacterial culture (*Giemsa*; lower right panel) identified by 16S PCR as *E* *. meningoseptica* species.
(TIFF)

Figure S4 **Efficient colonization of *E* *. meningoseptica* in mosquito tissues and F1-larvae.** *E* *. meningoseptica* fed to germ-free females shows massive colonization of the mosquito gut (*E.m*) compared to common microbial flora in conventionally-reared mosquitoes (CR). F1 larvae from E.m-reconstituted females show high abundance of *E* *. meningoseptica* distinct from microbial flora from CR larvae, as evidenced on plating. Randomly picked colonies on E.m plates were all identified as *E* *. meningoseptica* .
(TIFF)

Figure S5 **Relative distribution of bacterial species in *Anopheles gambiae* larvae reared with** ***E* *. meningoseptica* -derived F1 larvae**. The bacteria species were determined to be closely related to *Asaia* *sp.* *,* *Cedecea* *sp.* *,* *Elizabethkingia* *sp.* *,* *Gibbsiella* *sp.* *,* *Klebsiella* *sp.* *,* *Plesiomonas* *sp.* *,* *Rahnella* *sp.* and *Serratia* *sp* . Relative distribution in percentage is shown per individual mosquito. Note that some samples (3,5,6,8) do not harbour detectable *Elizabethkingia* colonies.
(TIFF)

## References

[1] Backhed F, Ley RE, Sonnenburg JL, Peterson DA, Gordon JI (2005) Host-bacterial mutualism in the human intestine. Science 307: 1915-1920. doi:10.1126/science.1104816. PubMed: 15790844. 15790844 10.1126/science.1104816

[2] Ley RE, Peterson DA, Gordon JI (2006) Ecological and evolutionary forces shaping microbial diversity in the human intestine. Cell 124: 837-848. doi:10.1016/j.cell.2006.02.017. PubMed: 16497592. 16497592 10.1016/j.cell.2006.02.017

[3] Pumpuni CB, Beier MS, Nataro JP, Guers LD, Davis JR (1993) Plasmodium falciparum: inhibition of sporogonic development in Anopheles stephensi by gram-negative bacteria. Exp Parasitol 77: 195-199. doi:10.1006/expr.1993.1076. PubMed: 8375488. 8375488 10.1006/expr.1993.1076

[4] Beier MS, Pumpuni CB, Beier JC, Davis JR (1994) Effects of para-aminobenzoic acid, insulin, and gentamicin on Plasmodium falciparum development in anopheline mosquitoes (Diptera: Culicidae). J Med Entomol 31: 561-565. PubMed: 7932602. 7932602 10.1093/jmedent/31.4.561

[5] Demaio J, Pumpuni CB, Kent M, Beier JC (1996) The midgut bacterial flora of wild Aedes triseriatus, Culex pipiens, and Psorophora columbiae mosquitoes. Am J Trop Med Hyg 54: 219-223. PubMed: 8619452. 8619452 10.4269/ajtmh.1996.54.219

[6] Pumpuni CB, Demaio J, Kent M, Davis JR, Beier JC (1996) Bacterial population dynamics in three anopheline species: the impact on Plasmodium sporogonic development. Am J Trop Med Hyg 54: 214-218. PubMed: 8619451. 8619451 10.4269/ajtmh.1996.54.214

[7] Lowenberger CA, Kamal S, Chiles J, Paskewitz S, Bulet P et al. (1999) Mosquito-Plasmodium interactions in response to immune activation of the vector. Exp Parasitol 91: 59-69. doi:10.1006/expr.1999.4350. PubMed: 9920043. 9920043 10.1006/expr.1999.4350

[8] Dong Y, Manfredini F, Dimopoulos G (2009) Implication of the mosquito midgut microbiota in the defense against malaria parasites. PLOS Pathog 5: e1000423 PubMed: 19424427. 19424427 10.1371/journal.ppat.1000423 PMC2673032

[9] Meister S, Agianian B, Turlure F, Relógio A, Morlais I et al. (2009) Anopheles gambiae PGRPLC-mediated defense against bacteria modulates infections with malaria parasites. PLOS Pathog 5: e1000542 PubMed: 19662170. 19662170 10.1371/journal.ppat.1000542 PMC2715215

[10] Noden BH, Vaughan JA, Pumpuni CB, Beier JC (2011) Mosquito ingestion of antibodies against mosquito midgut microbiota improves conversion of ookinetes to oocysts for Plasmodium falciparum, but not P. yoelii. Parasitol Int 60: 440-446. doi:10.1016/j.parint.2011.07.007. PubMed: 21763778. 21763778 10.1016/j.parint.2011.07.007 PMC3209551

[11] Cirimotich CM, Dong Y, Clayton AM, Sandiford SL, Souza-Neto JA et al. (2011) Natural microbe-mediated refractoriness to Plasmodium infection in Anopheles gambiae. Science 332: 855-858. doi:10.1126/science.1201618. PubMed: 21566196. 21566196 10.1126/science.1201618 PMC4154605

[12] Boissière A, Tchioffo MT, Bachar D, Abate L, Marie A et al. (2012) Midgut Microbiota of the Malaria Mosquito Vector Anopheles gambiae and Interactions with Plasmodium falciparum Infection. PLOS Pathog 8: e1002742 PubMed: 22693451. 22693451 10.1371/journal.ppat.1002742 PMC3364955

[13] Dimopoulos G, Seeley D, Wolf A, Kafatos FC (1998) Malaria infection of the mosquito Anopheles gambiae activates immune-responsive genes during critical transition stages of the parasite life cycle. EMBO J 17: 6115-6123. doi:10.1093/emboj/17.21.6115. PubMed: 9799221. 9799221 10.1093/emboj/17.21.6115 PMC1170938

[14] Han YS, Thompson J, Kafatos FC, Barillas-Mury C (2000) Molecular interactions between Anopheles stephensi midgut cells and Plasmodium berghei: the time bomb theory of ookinete invasion of mosquitoes. EMBO J 19: 6030-6040. doi:10.1093/emboj/19.22.6030. PubMed: 11080150. 11080150 10.1093/emboj/19.22.6030 PMC305834

[15] Alavi Y, Arai M, Mendoza J, Tufet-Bayona M, Sinha R et al. (2003) The dynamics of interactions between Plasmodium and the mosquito: a study of the infectivity of Plasmodium berghei and Plasmodium gallinaceum, and their transmission by Anopheles stephensi, Anopheles gambiae and Aedes aegypti. Int J Parasitol 33: 933-943. doi:10.1016/S0020-7519(03)00112-7. PubMed: 12906877. 12906877 10.1016/s0020-7519(03)00112-7

[16] Vlachou D, Schlegelmilch T, Christophides GK, Kafatos FC (2005) Functional genomic analysis of midgut epithelial responses in Anopheles during Plasmodium invasion. Curr Biol 15: 1185-1195. doi:10.1016/j.cub.2005.06.044. PubMed: 16005290. 16005290 10.1016/j.cub.2005.06.044

[17] Lindh JM, Terenius O, Faye I (2005) 16S rRNA gene-based identification of midgut bacteria from field-caught Anopheles gambiae sensu lato and A. funestus mosquitoes reveals new species related to known insect symbionts. Appl Environ Microbiol 71: 7217-7223. doi:10.1128/AEM.71.11.7217-7223.2005. PubMed: 16269761. 16269761 10.1128/AEM.71.11.7217-7223.2005 PMC1287614

[18] Rani A, Sharma A, Rajagopal R, Adak T, Bhatnagar RK (2009) Bacterial diversity analysis of larvae and adult midgut microflora using culture-dependent and culture-independent methods in lab-reared and field-collected Anopheles stephensi-an Asian malarial vector. BMC Microbiol 9: 96. doi:10.1186/1471-2180-9-96. PubMed: 19450290. 19450290 10.1186/1471-2180-9-96 PMC2698833

[19] Favia G, Ricci I, Damiani C, Raddadi N, Crotti E et al. (2007) Bacteria of the genus Asaia stably associate with Anopheles stephensi, an Asian malarial mosquito vector. Proc Natl Acad Sci U S A 104: 9047-9051. doi:10.1073/pnas.0610451104. PubMed: 17502606. 17502606 10.1073/pnas.0610451104 PMC1885625

[20] Lindh JM, Borg-Karlson AK, Faye I (2008) Transstadial and horizontal transfer of bacteria within a colony of Anopheles gambiae (Diptera: Culicidae) and oviposition response to bacteria-containing water. Acta Trop 107: 242-250. doi:10.1016/j.actatropica.2008.06.008. PubMed: 18671931. 18671931 10.1016/j.actatropica.2008.06.008

[21] Damiani C, Ricci I, Crotti E, Rossi P, Rizzi A et al. (2010) Mosquito-bacteria symbiosis: the case of Anopheles gambiae and Asaia. Microb Ecol 60: 644-654. doi:10.1007/s00248-010-9704-8. PubMed: 20571792. 20571792 10.1007/s00248-010-9704-8

[22] Kim KK, Kim MK, Lim JH, Park HY, Lee ST (2005) Transfer of Chryseobacterium meningosepticum and Chryseobacterium miricola to Elizabethkingia gen. nov. as Elizabethkingia meningoseptica comb. nov. and Elizabethkingia miricola comb. nov. Int J Syst Evol Microbiol 55: 1287-1293. doi:10.1099/ijs.0.63541-0. PubMed: 15879269. 15879269 10.1099/ijs.0.63541-0

[23] Kämpfer P, Matthews H, Glaeser SP, Martin K, Lodders N et al. (2011) Elizabethkingia anophelis sp. nov., isolated from the midgut of the mosquito Anopheles gambiae. Int J Syst Evol Microbiol 61: 2670-2675. doi:10.1099/ijs.0.026393-0. PubMed: 21169462. 21169462 10.1099/ijs.0.026393-0

[24] King EO (1959) Studies on a group of previously unclassified bacteria associated with meningitis in infants. Am J Clin Pathol 31: 241-247. PubMed: 13637033. 13637033 10.1093/ajcp/31.3.241

[25] Dias M, Fernandes A, Furtado Z (2012) Case series: elizabethkingia meningosepticum. J Clin Diagn Res 6: 1550-1551. PubMed: 23285454. 23285454 10.7860/JCDR/2012/4076.2557 PMC3527794

[26] Wang Y, Gilbreath TM 3rd, Kukutla P, Yan G, Xu J (2011) Dynamic gut microbiome across life history of the malaria mosquito Anopheles gambiae in Kenya. PLOS ONE 6: e24767. doi:10.1371/journal.pone.0024767. PubMed: 21957459. 21957459 10.1371/journal.pone.0024767 PMC3177825

[27] Ngwa CJ, Glöckner V, Abdelmohsen UR, Scheuermayer M, Fischer R et al. (2013) 16S rRNA gene-based identification of Elizabethkingia meningoseptica (Flavobacteriales: Flavobacteriaceae) as a dominant midgut bacterium of the Asian malaria vector Anopheles stephensi (Dipteria: Culicidae) with antimicrobial activities. J Med Entomol 50: 404-414. doi:10.1603/ME12180. PubMed: 23540130. 23540130 10.1603/me12180

[28] Nappi AJ (1973) Hemocytic changes associated with the encapsulation and melanization of some insect parasites. Exp Parasitol 33: 285-302. doi:10.1016/0014-4894(73)90034-9. PubMed: 4196234. 4196234 10.1016/0014-4894(73)90034-9

[29] Li J, Hodgeman BA, Christensen BM (1996) Involvement of peroxidase in chorion hardening in Aedes aegypti. Insect Biochem Mol Biol 26: 309-317. doi:10.1016/0965-1748(95)00099-2. PubMed: 8900599. 8900599 10.1016/0965-1748(95)00099-2

[30] Lavine MD, Strand MR (2002) Insect hemocytes and their role in immunity. Insect Biochem Mol Biol 32: 1295-1309. doi:10.1016/S0965-1748(02)00092-9. PubMed: 12225920. 12225920 10.1016/s0965-1748(02)00092-9

[31] Christensen BM, Li J, Chen CC, Nappi AJ (2005) Melanization immune responses in mosquito vectors. Trends Parasitol 21: 192-199. doi:10.1016/j.pt.2005.02.007. PubMed: 15780842. 15780842 10.1016/j.pt.2005.02.007

[32] Tang H (2009) Regulation and function of the melanization reaction in Drosophila. Fly (Austin) 3: 105-111. doi:10.4161/fly.3.1.7747. PubMed: 19164947. 19164947 10.4161/fly.3.1.7747

[33] Cerenius L, Söderhäll K (2004) The prophenoloxidase-activating system in invertebrates. Immunol Rev 198: 116-126. doi:10.1111/j.0105-2896.2004.00116.x. PubMed: 15199959. 15199959 10.1111/j.0105-2896.2004.00116.x

[34] Blandin S, Moita LF, Köcher T, Wilm M, Kafatos FC et al. (2002) Reverse genetics in the mosquito Anopheles gambiae: targeted disruption of the Defensin gene. EMBO Rep 3: 852-856. doi:10.1093/embo-reports/kvf180. PubMed: 12189180. 12189180 10.1093/embo-reports/kvf180 PMC1084233

[35] Schnitger AK, Kafatos FC, Osta MA (2007) The melanization reaction is not required for survival of Anopheles gambiae mosquitoes after bacterial infections. J Biol Chem 282: 21884-21888. doi:10.1074/jbc.M701635200. PubMed: 17537726. 17537726 10.1074/jbc.M701635200

[36] Harshbarger JC, Taylor RL (1968) Neoplasms of Insects. Annu Rev Entomol 13: 159-190. doi:10.1146/annurev.en.13.010168.001111.

[37] Sugumaran M (1991) Molecular mechanisms for mammalian melanogenesis. Comparison with insect cuticular sclerotization. FEBS Lett 295: 233-239. doi:10.1016/0014-5793(91)81431-7. PubMed: 1765160. 1765160 10.1016/0014-5793(91)81431-7

[38] Gateff E (1978) Malignant neoplasms of genetic origin in Drosophila melanogaster. Science 200: 1448-1459. doi:10.1126/science.96525. PubMed: 96525. 96525 10.1126/science.96525

[39] Rodriguez A, Zhou Z, Tang ML, Meller S, Chen J et al. (1996) Identification of immune system and response genes, and novel mutations causing melanotic tumor formation in Drosophila melanogaster. Genetics 143: 929-940. PubMed: 8725239. 8725239 10.1093/genetics/143.2.929 PMC1207349

[40] Minakhina S, Steward R (2006) Melanotic mutants in Drosophila: pathways and phenotypes. Genetics 174: 253-263. doi:10.1534/genetics.106.061978. PubMed: 16816412. 16816412 10.1534/genetics.106.061978 PMC1569781

[41] Avet-Rochex A, Boyer K, Polesello C, Gobert V, Osman D et al. (2010) An in vivo RNA interference screen identifies gene networks controlling Drosophila melanogaster blood cell homeostasis. BMC Dev Biol 10: 65. doi:10.1186/1471-213X-10-65. PubMed: 20540764. 20540764 10.1186/1471-213X-10-65 PMC2891661

[42] Park JW, Kim CH, Kim JH, Je BR, Roh KB et al. (2007) Clustering of peptidoglycan recognition protein-SA is required for sensing lysine-type peptidoglycan in insects. Proc Natl Acad Sci U S A 104: 6602-6607. doi:10.1073/pnas.0610924104. PubMed: 17409189. 17409189 10.1073/pnas.0610924104 PMC1871832

[43] Paddibhatla I, Lee MJ, Kalamarz ME, Ferrarese R, Govind S (2010) Role for sumoylation in systemic inflammation and immune homeostasis in Drosophila larvae. PLOS Pathog 6: e1001234 PubMed: 21203476. 21203476 10.1371/journal.ppat.1001234 PMC3009591

[44] Wang S, Ghosh AK, Bongio N, Stebbings KA, Lampe DJ et al. (2012) Fighting malaria with engineered symbiotic bacteria from vector mosquitoes. Proc Natl Acad Sci U S A 109: 12734-12739. doi:10.1073/pnas.1204158109. PubMed: 22802646. 22802646 10.1073/pnas.1204158109 PMC3412027

[45] Lee WJ (2008) Bacterial-modulated signaling pathways in gut homeostasis. Sci Signal 1: e24. 10.1126/stke.121pe24 18506033

[46] Ryu JH, Kim SH, Lee HY, Bai JY, Nam YD et al. (2008) Innate immune homeostasis by the homeobox gene caudal and commensal-gut mutualism in Drosophila. Science 319: 777-782. doi:10.1126/science.1149357. PubMed: 18218863. 18218863 10.1126/science.1149357

[47] Shin SC, Kim SH, You H, Kim B, Kim AC et al. (2011) Drosophila microbiome modulates host developmental and metabolic homeostasis via insulin signaling. Science 334: 670-674. doi:10.1126/science.1212782. PubMed: 22053049. 22053049 10.1126/science.1212782

[48] Storelli G, Defaye A, Erkosar B, Hols P, Royet J et al. (2011) Lactobacillus plantarum promotes Drosophila systemic growth by modulating hormonal signals through TOR-dependent nutrient sensing. Cell Metab 14: 403-414. doi:10.1016/j.cmet.2011.07.012. PubMed: 21907145. 21907145 10.1016/j.cmet.2011.07.012

[49] Chouaia B, Rossi P, Epis S, Mosca M, Ricci I et al. (2012) Delayed larval development in Anopheles mosquitoes deprived of Asaia bacterial symbionts. BMC Microbiol 12 Suppl 1: S2. doi:10.1186/1471-2180-12-2. PubMed: 22375964. 22375964 10.1186/1471-2180-12-S1-S2 PMC3287513

[50] Harrison G (1978) Mosquitoes, malaria and man: a history of hostilities since 1880: New York E.P.

[51] WHO (2011) World malaria report 2011.

[52] Curtis CF (2002) Restoration of malaria control in the Madagascar highlands by DDT spraying. Am J Trop Med Hyg 66: 1 PubMed: 12135257. 12135257 10.4269/ajtmh.2002.66.1

[53] Pates H, Curtis C (2005) Mosquito behavior and vector control. Annu Rev Entomol 50: 53-70. doi:10.1146/annurev.ento.50.071803.130439. PubMed: 15355233. 15355233 10.1146/annurev.ento.50.071803.130439

[54] Lindblade KA, Gimnig JE, Kamau L, Hawley WA, Odhiambo F et al. (2006) Impact of sustained use of insecticide-treated bednets on malaria vector species distribution and culicine mosquitoes. J Med Entomol 43: 428-432. doi:10.1603/0022-2585(2006)043[0428:IOSUOI]2.0.CO;2. PubMed: 16619629. 16619629 10.1603/0022-2585(2006)043[0428:iosuoi]2.0.co;2

[55] WHO (1982) Lutte biologique contre les vecteurs de maladies. Série Rapports TechniqueS 679.

[56] Hemingway J, Ranson H (2000) Insecticide resistance in insect vectors of human disease. Annu Rev Entomol 45: 371-391. doi:10.1146/annurev.ento.45.1.371. PubMed: 10761582. 10761582 10.1146/annurev.ento.45.1.371

[57] Farenhorst M, Mouatcho JC, Kikankie CK, Brooke BD, Hunt RH et al. (2009) Fungal infection counters insecticide resistance in African malaria mosquitoes. Proc Natl Acad Sci U S A 106: 17443-17447. doi:10.1073/pnas.0908530106. PubMed: 19805146. 19805146 10.1073/pnas.0908530106 PMC2762667

[58] Tetreau G, Chandor-Proust A, Faucon F, Stalinski R, Akhouayri I et al. (2013) Contrasting patterns of tolerance between chemical and biological insecticides in mosquitoes exposed to UV-A. Aquat Toxicol, 140-141: 389–97. PubMed: 23911355. 23911355 10.1016/j.aquatox.2013.07.004

[59] Durvasula RV, Gumbs A, Panackal A, Kruglov O, Aksoy S et al. (1997) Prevention of insect-borne disease: an approach using transgenic symbiotic bacteria. Proc Natl Acad Sci U S A 94: 3274-3278. doi:10.1073/pnas.94.7.3274. PubMed: 9096383. 9096383 10.1073/pnas.94.7.3274 PMC20359

[60] Beard CB, Durvasula RV, Richards FF (1998) Bacterial symbiosis in arthropods and the control of disease transmission. Emerg Infect Dis 4: 581-591. doi:10.3201/eid0404.980408. PubMed: 9866734. 9866734 10.3201/eid0404.980408 PMC2640264

[61] Favia G, Ricci I, Marzorati M, Negri I, Alma A et al. (2008) Bacteria of the genus Asaia: a potential paratransgenic weapon against malaria. Adv Exp Med Biol 627: 49-59. doi:10.1007/978-0-387-78225-6_4. PubMed: 18510013. 18510013 10.1007/978-0-387-78225-6_4

[62] Kambris Z, Cook PE, Phuc HK, Sinkins SP (2009) Immune activation by life-shortening Wolbachia and reduced filarial competence in mosquitoes. Science 326: 134-136. doi:10.1126/science.1177531. PubMed: 19797660. 19797660 10.1126/science.1177531 PMC2867033

[63] Bisi DC, Lampe DJ (2011) Secretion of anti-Plasmodium effector proteins from a natural Pantoea agglomerans isolate by using PelB and HlyA secretion signals. Appl Environ Microbiol 77: 4669-4675. doi:10.1128/AEM.00514-11. PubMed: 21602368. 21602368 10.1128/AEM.00514-11 PMC3127683

[64] Ricci I, Mosca M, Valzano M, Damiani C, Scuppa P et al. (2011) Different mosquito species host Wickerhamomyces anomalus (Pichia anomala): perspectives on vector-borne diseases symbiotic control. Antonie Van Leeuwenhoek 99: 43-50. doi:10.1007/s10482-010-9532-3. PubMed: 21113816. 21113816 10.1007/s10482-010-9532-3

[65] Cirimotich CM, Ramirez JL, Dimopoulos G (2011) Native microbiota shape insect vector competence for human pathogens. Cell Host Microbe 10: 307-310. doi:10.1016/j.chom.2011.09.006. PubMed: 22018231. 22018231 10.1016/j.chom.2011.09.006 PMC3462649

[66] Bian G, Joshi D, Dong Y, Lu P, Zhou G et al. (2013) Wolbachia invades Anopheles stephensi populations and induces refractoriness to Plasmodium infection. Science 340: 748-751. doi:10.1126/science.1236192. PubMed: 23661760. 23661760 10.1126/science.1236192

[67] Frank JA, Reich CI, Sharma S, Weisbaum JS, Wilson BA et al. (2008) Critical evaluation of two primers commonly used for amplification of bacterial 16S rRNA genes. Appl Environ Microbiol 74: 2461-2470. doi:10.1128/AEM.02272-07. PubMed: 18296538. 18296538 10.1128/AEM.02272-07 PMC2293150

