# Supplementary figures and images for: Melanotic Pathology and Vertical Transmission of the Gut Commensal Elizabethkingia meningoseptica in the Major Malaria Vector Anopheles gambiae
Source: PLoS One. 2013 Oct 1;8(10):e77619. doi: 10.1371/journal.pone.0077619 (PMC3788111; doi:10.1371/journal.pone.0077619)

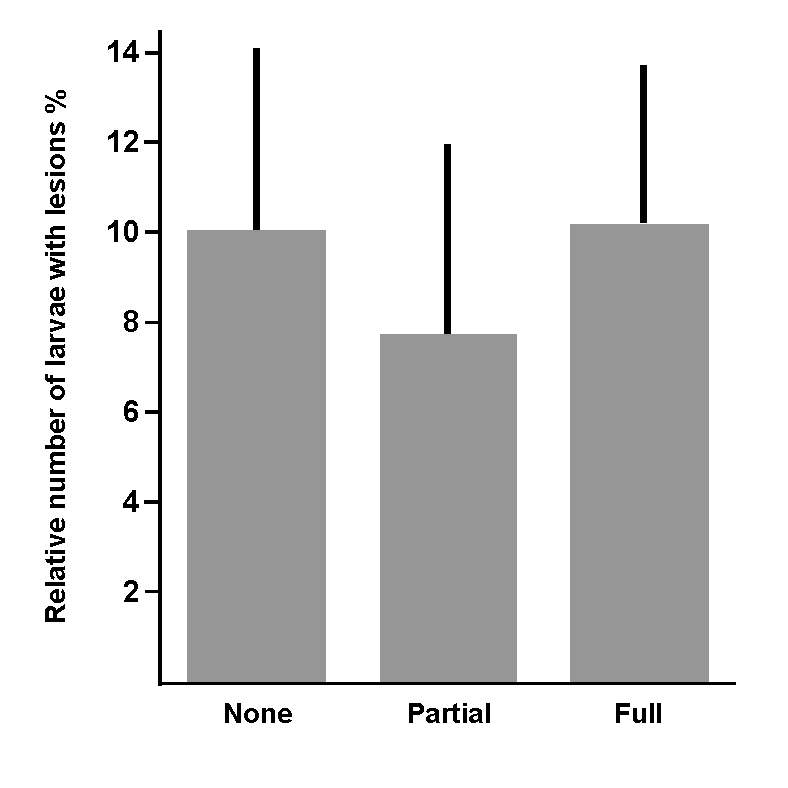

Supplement: Figure S1 — Antibiotic treatment does not affect recovery of lesion-bearing larvae. Antibiotic solutions were applied to larval stages 7-8 days post-egg laying (n=300), from 3 biological replicates. Larval instars harbouring melanotic lesions were counted daily until pupation (None). Normal diet without antibiotics; (Partial) Intermediate antibiotic concentration [5mL streptomycin/Penicillin (200X dilution in 1L rearing water)]; (Full) Concentrated antibiotic treatment [25mL Pen/Strep (40X dilution in 1L rearing water)]. Mean value of relative numbers of larvae showing lesions is represented as bar with standard for each condition. (TIFF) [file pone.0077619.s001.tiff]

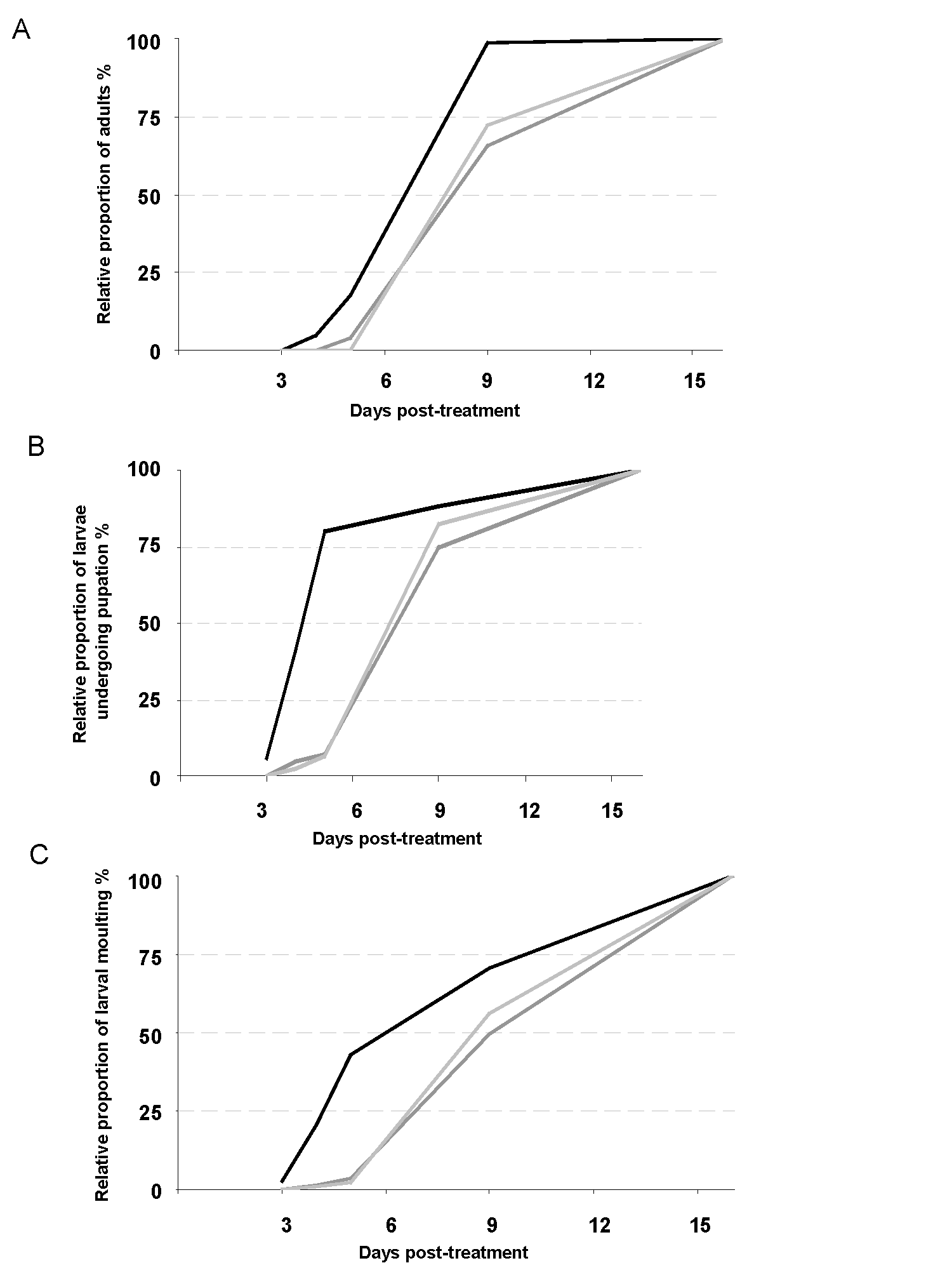

Supplement: Figure S2 — Mosquito developmental delay after antibiotic treatment. Antibiotic treatment was applied to larval stages as described in Figure S1 (black line). Control normal diet without antibiotics; (dark grey) Partial treatment; (light grey) Full treatment. (A) Adult emergence; Note that most adults appeared at day 9 in control, whereas antibiotic-treated groups showed 65-70% emergence on the same day. (B) Pupation peaks at day 4 in normal diet, whereas a 5 days-delay occurred in treated groups for pupation to proceed. (C) Larval developmental rate; a severe developmental slow-down appeared in treated larvae, since 50% of all moulting (from Ist to IVth instars) occurred 4-5 days later compared to controls, showing the strong impact of antibiotics at the larval feeding stages. (TIFF) [file pone.0077619.s002.tiff]

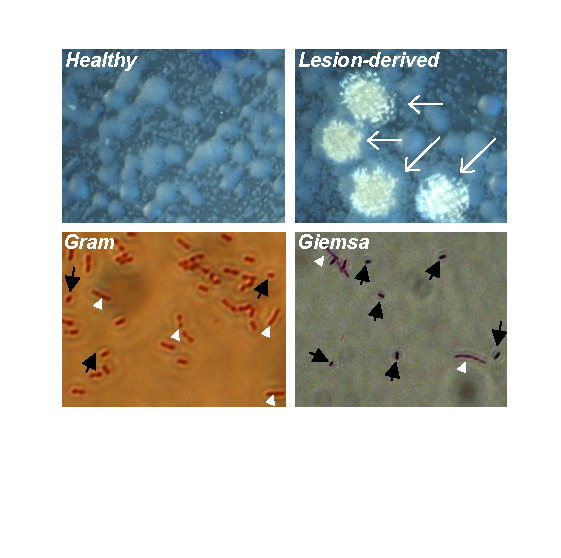

Supplement: Figure S3 — Identification of E . meningoseptica . Micrographs of ampicillin-LB plates with bacterial colonies derived from control (Healthy; top left panel) and lesion-affected (Lesion-derived; top right panel) fat body tissues from larvae. Note major bacterial colonies (white arrows) specifically recovered from lesion-affected tissues. These colonies were Gram-stained-negative rod-shaped cells (Gram; lower left panel) with individual (black arrows) and dividing cells (white arrowheads) visible on Gram- and Giemsa-stained bacterial culture (Giemsa; lower right panel) identified by 16S PCR as E . meningoseptica species. (TIFF) [file pone.0077619.s003.tiff]

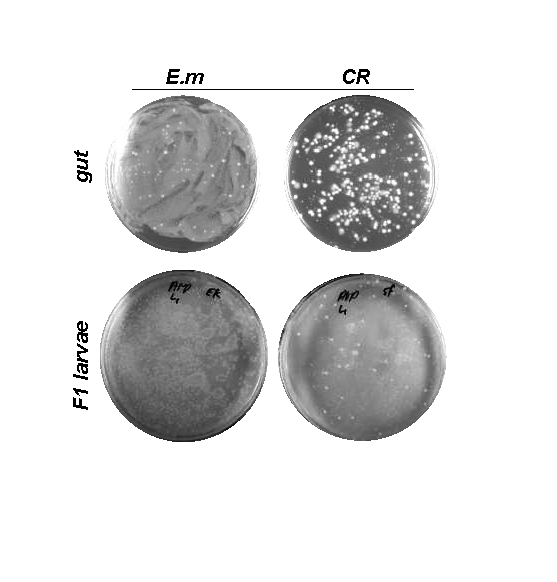

Supplement: Figure S4 — Efficient colonization of E . meningoseptica in mosquito tissues and F1-larvae. E . meningoseptica fed to germ-free females shows massive colonization of the mosquito gut (E.m) compared to common microbial flora in conventionally-reared mosquitoes (CR). F1 larvae from E.m-reconstituted females show high abundance of E . meningoseptica distinct from microbial flora from CR larvae, as evidenced on plating. Randomly picked colonies on E.m plates were all identified as E . meningoseptica . (TIFF) [file pone.0077619.s004.tiff]

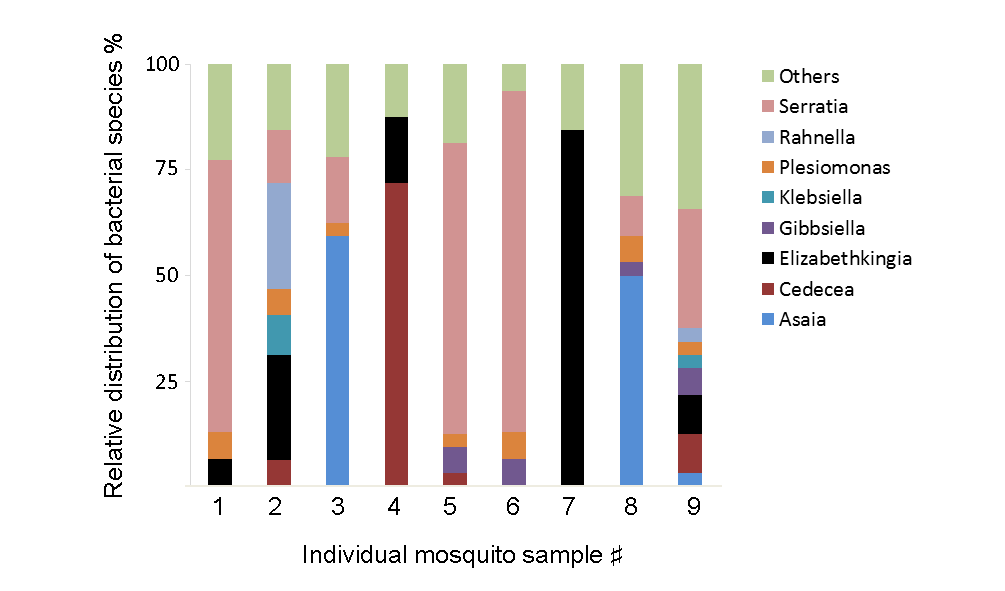

Supplement: Figure S5 — Relative distribution of bacterial species in Anopheles gambiae larvae reared with E . meningoseptica -derived F1 larvae. The bacteria species were determined to be closely related to Asaia sp. , Cedecea sp. , Elizabethkingia sp. , Gibbsiella sp. , Klebsiella sp. , Plesiomonas sp. , Rahnella sp. and Serratia sp . Relative distribution in percentage is shown per individual mosquito. Note that some samples (3,5,6,8) do not harbour detectable Elizabethkingia colonies. (TIFF) [file pone.0077619.s005.tiff]
